# Pharmacophenotype identification of intensive care unit medications using unsupervised cluster analysis of the ICURx common data model

**DOI:** 10.1186/s13054-023-04437-2

**Published:** 2023-05-02

**Authors:** Andrea Sikora, Alireza Rafiei, Milad Ghiasi Rad, Kelli Keats, Susan E. Smith, John W. Devlin, David J. Murphy, Brian Murray, Rishikesan Kamaleswaran, MRC-ICU Investigator Team

**Affiliations:** 1 Department of Clinical and Administrative Pharmacy, University of Georgia College of Pharmacy, Augusta, GA USA; 2 Department of Computer Science and Informatics, Emory University, Atlanta, GA USA; 3 Department of Electrical and Computer Engineering, Georgia Institute of Technology, Atlanta, GA USA; 4 Department of Pharmacy, Augusta University Medical Center, Augusta, GA USA; 5 Northeastern University School of Pharmacy, Boston, MA USA; 6 Brigham and Women’s Hospital, Division of Pulmonary and Critical Care Medicine, Boston, MA USA; 7 Division of Pulmonary, Allergy, Critical Care and Sleep Medicine, Emory University, Atlanta, GA USA; 8 Department of Pharmacy, University of North Carolina Medical Center, Chapel Hill, NC USA; 9 Department of Biomedical Informatics, Emory University School of Medicine, Atlanta, GA USA; 10 Department of Biomedical Engineering, Georgia Institute of Technology, Atlanta, GA USA

**Keywords:** Machine learning, Unsupervised learning, Cluster analysis, Intensive care unit, Critical care, Medications, Outcomes

## Abstract

**Background:**

Identifying patterns within ICU medication regimens may help artificial intelligence algorithms to better predict patient outcomes; however, machine learning methods incorporating medications require further development, including standardized terminology. The Common Data Model for Intensive Care Unit (ICU) Medications (CDM-ICURx) may provide important infrastructure to clinicians and researchers to support artificial intelligence analysis of medication-related outcomes and healthcare costs. Using an unsupervised cluster analysis approach in combination with this common data model, the objective of this evaluation was to identify novel patterns of medication clusters (termed ‘pharmacophenotypes’) correlated with ICU adverse events (e.g., fluid overload) and patient-centered outcomes (e.g., mortality).

**Methods:**

This was a retrospective, observational cohort study of 991 critically ill adults. To identify pharmacophenotypes, unsupervised machine learning analysis with automated feature learning using restricted Boltzmann machine and hierarchical clustering was performed on the medication administration records of each patient during the first 24 h of their ICU stay. Hierarchical agglomerative clustering was applied to identify unique patient clusters. Distributions of medications across pharmacophenotypes were described, and differences among patient clusters were compared using signed rank tests and Fisher's exact tests, as appropriate.

**Results:**

A total of 30,550 medication orders for the 991 patients were analyzed; five unique patient clusters and six unique pharmacophenotypes were identified. For patient outcomes, compared to patients in Clusters 1 and 3, patients in Cluster 5 had a significantly shorter duration of mechanical ventilation and ICU length of stay (*p* < 0.05); for medications, Cluster 5 had a higher distribution of Pharmacophenotype 1 and a smaller distribution of Pharmacophenotype 2, compared to Clusters 1 and 3. For outcomes, patients in Cluster 2, despite having the highest severity of illness and greatest medication regimen complexity, had the lowest overall mortality; for medications, Cluster 2 also had a comparably higher distribution of Pharmacophenotype 6.

**Conclusion:**

The results of this evaluation suggest that patterns among patient clusters and medication regimens may be observed using empiric methods of unsupervised machine learning in combination with a common data model. These results have potential because while phenotyping approaches have been used to classify heterogenous syndromes in critical illness to better define treatment response, the entire medication administration record has not been incorporated in those analyses. Applying knowledge of these patterns at the bedside requires further algorithm development and clinical application but may have the future potential to be leveraged in guiding medication-related decision making to improve treatment outcomes.

**Supplementary Information:**

The online version contains supplementary material available at 10.1186/s13054-023-04437-2.

## Introduction

With over 20,000 Federal Food and Drug Administration-approved medication products that can be ordered and administered in a myriad of different ways, the medication regimens of critically ill patients have a nearly infinite number of permutations [1, 2]. Because adverse drug events occur more frequently in intensive care unit (ICU) than non-ICU patients and confer significantly increased mortality risks in the form of ICU complications, management of these complex medication regimens is essential to optimizing ICU patient safety and outcomes [3, 4].

Efforts to characterize the heterogeneous nature of ICU medication regimens and how these medications act in concert with each other and in the context of critical illness have only just begun [5–9]. Medication regimen heterogeneity parallels the challenging disease heterogeneity of critical illness [10, 11]. Phenotyping is a novel concept starting to be used to characterize between-patient heterogeneity for common ICU conditions like sepsis/shock and acute respiratory distress syndrome (ARDS) [12–19]. Phenotyping using machine learning has demonstrated the potential to be a powerful methodology to handle Big Data generated by critically ill patients for phenotype delineation and prediction of adverse events [12–19], and identifying these sub-patterns within well-known (but often poorly mechanized) disease states has shown treatment specific responses patterns not previously appreciated in large-scale studies using traditional methodology [20–23]. The application of a phenotypic approach to ICU medication use may reveal novel response patterns that can be applied to improve medication safety and efficacy: for example, while certain combinations of medications are widely recognized to be associated with ICU complications (e.g., opioids and benzodiazepines with mechanical ventilation duration), a possibility exists that combinations of medications not commonly recognized by the clinical eye have a similar risk profile. However, the associated methodology and common data model infrastructure have not been previously developed for this type of exploration.

A significant challenge facing the use of artificial intelligence to explore ICU medication use is the lack of common data models to both standardize ontology and assist machines in interpreting medication orders and the nuances of this therapy. Existing common data models oversimplify the complex, high-risk nature of ICU medications that results in potentially life-threatening adverse drug events affecting 1.5 million critically ill adults in the USA annually [4, 24, 25]. The absence of a specific common data model for ICU medications prohibits the ability to interrogate electronic health records (EHRs) to predict and prevent such adverse drug events. For example, the antibiotic cefepime might be ordered as cefepime 2 g every 12 h or cefepime 1 g every 24 h. While these dosing schema are different, the adverse effect profile (including allergy risk) is likely the same for both. Moreover, renal function plays an important role for interpreting ICU drug dose and frequency. While cefepime is typically dosed at 2 g every 12 h, in renal failure it is dosed 1 g every 24 h unless renal failure is being support with continuous renal replacement therapy (CRRT) when the usual cefepime dose increases to 2 g every 8 h. Without knowing a patient’s renal function and use of CRRT, it is not possible to know if cefepime 1 g every 24 h regimen is appropriate or an underdose in the magnitude of 4-to-6-fold, which could result in a potentially fatal condition of under-treated sepsis. Notably, this type of error would be difficult to catch with traditional dose-checking software, as both are acceptable dosing schema by drug library standards.

The objective of this study was to explore the presence of novel medication patterns (termed ‘pharmacophenotypes’) correlated with ICU adverse events and patient-centered outcomes in critically ill adults using an unsupervised learning approach that employed an ICU medication focused common data model.

## Methods

### Study sample

The study cohort included patients ≥ 18 years old who were admitted ≥ 24 h to a medical, surgical, neuroscience, cardiac, or burn ICU at the University of North Carolina Health System between October 2015 and October 2020. Only a patient’s index ICU admission was used for the analysis, and patients with restrictions to care (e.g., comfort care) in the first 24 h were excluded. Study patients were identified through the University of North Carolina Health System EHR system (Epic Systems, Verona, WI). All de-identified patient information was extracted from the Carolina Data Warehouse by a trained in-house data analyst. The institutional review board at The University of Georgia approved this study and included a waiver of consent (PROJECT00002652).

The EHR was queried for patient demographics, medication administration record (MAR) information, and patient outcomes, including common ICU complications. Patient demographics consisted of age, sex, admission diagnosis, ICU type, Acute Physiology and Chronic Health Evaluation II (APACHE II) score at 24 h, and medication regimen complexity-intensive care unit (MRC-ICU) score at 24 h. MRC-ICU is a previously validated score that quantifies the complexity of prescribed medications in the ICU and was included in this analysis as a means of summarizing high-risk, narrow therapeutic index medications commonly associated with need for increased monitoring as well as ICU complications [1, 6–8, 26–31]. MAR information included drug, dose, route, duration, and timing of administration in the first 24 h of the ICU stay. Patient outcomes included mortality, hospital length of stay, delirium occurrence (defined by Confusion Assessment Method for the ICU [CAM-ICU] positive score), duration of mechanical ventilation, duration of vasopressor use, and acute kidney injury (defined by the presence of renal replacement therapy or a serum creatinine greater than 1.5 times baseline). For each patient, a binary value of 1 was assigned to indicate that the patient received a particular drug order, which consisted of drug, dose, strength, and formulation/route. For patient outcomes, the labels for categorical features were relabeled as numeric values. In the cases of unknown or missing entities, they were counted as absences.

### Unsupervised learning approach

#### Medication clustering

To identify medication clusters (or pharmacophenotypes), principal component analysis was first performed on the processed, high-dimensional, binary medication dataset. Principal component analysis is a dimensionality-reduction technique that transforms high-dimension datasets into lower-dimension while retaining their properties [32]. Principal component analysis can increase the interpretability of the data by creating new variables to maximize variance. Every 600 unique medications were considered an independent variable, and the optimal number of principal components was chosen after visualizing the explained variance against principal component numbers. In this regard, 150 was selected for the number of principal components to maintain a sufficient amount of variance (more than 70%) in the data while reducing the dimensionality by a quarter.

The restricted Boltzmann machine was then employed to enrich the latent feature space using a hierarchical clustering algorithm input to generate automated features, which can support pharmacophenotype evaluation. Restricted Boltzmann machine is a generative two-layered neural network with one hidden layer and one visible layer [33]. This undirected model aims to discover the joint probability distribution, which can maximize the log-likelihood function to learn the complex internal representations of input variables [34]. For pharmacophenotype identification, restricted Boltzmann machine was used to learn the principal component analysis results’ unsupervised feature abstractions (or latent factors). In this way, restricted Boltzmann machine aimed to discover the relational nature of medication assignments based on each patient's medication co-occurrence. Using 5000 training epochs, restricted Boltzmann machine learned the activation pattern of every single hidden unit for clustering. Of note, every medication is considered an independent node in the visible layer, and connections activated to the hidden layer represent the pharmacophenotype assignment. For instance, if the connection of ‘Acyclovir 500 mg IVPB in 250 ml’ medication (from the visible layer) and Pharmacophenotype 2 (from the hidden layer) was activated, ‘Acyclovir 500 mg IVPB in 250 ml’ medication would be assigned to Pharmacophenotype 2. After assigning medications (visible layer) to each pharmacophenotype (hidden layer), medications that were not assigned to any of the five pharmacophenotype (never activated to one of the five hidden neurons) were grouped as Pharmacophenotype 6.

#### Patient clustering

To cluster patients, principal component analysis was applied on the processed, high-dimensional, binary medication dataset followed by normalization and agglomerative clustering.

#### Normalized medication cluster distribution

Since a single patient may be exposed to multiple medications over the course of their ICU stay, a frequency table was constructed to enumerate the count of each observation over time. This frequency table was normalized so that it considered the total number of medications that were administered to each patient and therefore generate a normalized pharmacophenotype distribution for each patient. The resulting normalized pharmacophenotype was used as a derived feature for the clustering of the patients.

#### Hierarchical agglomerative clustering

**Fig. 1:**
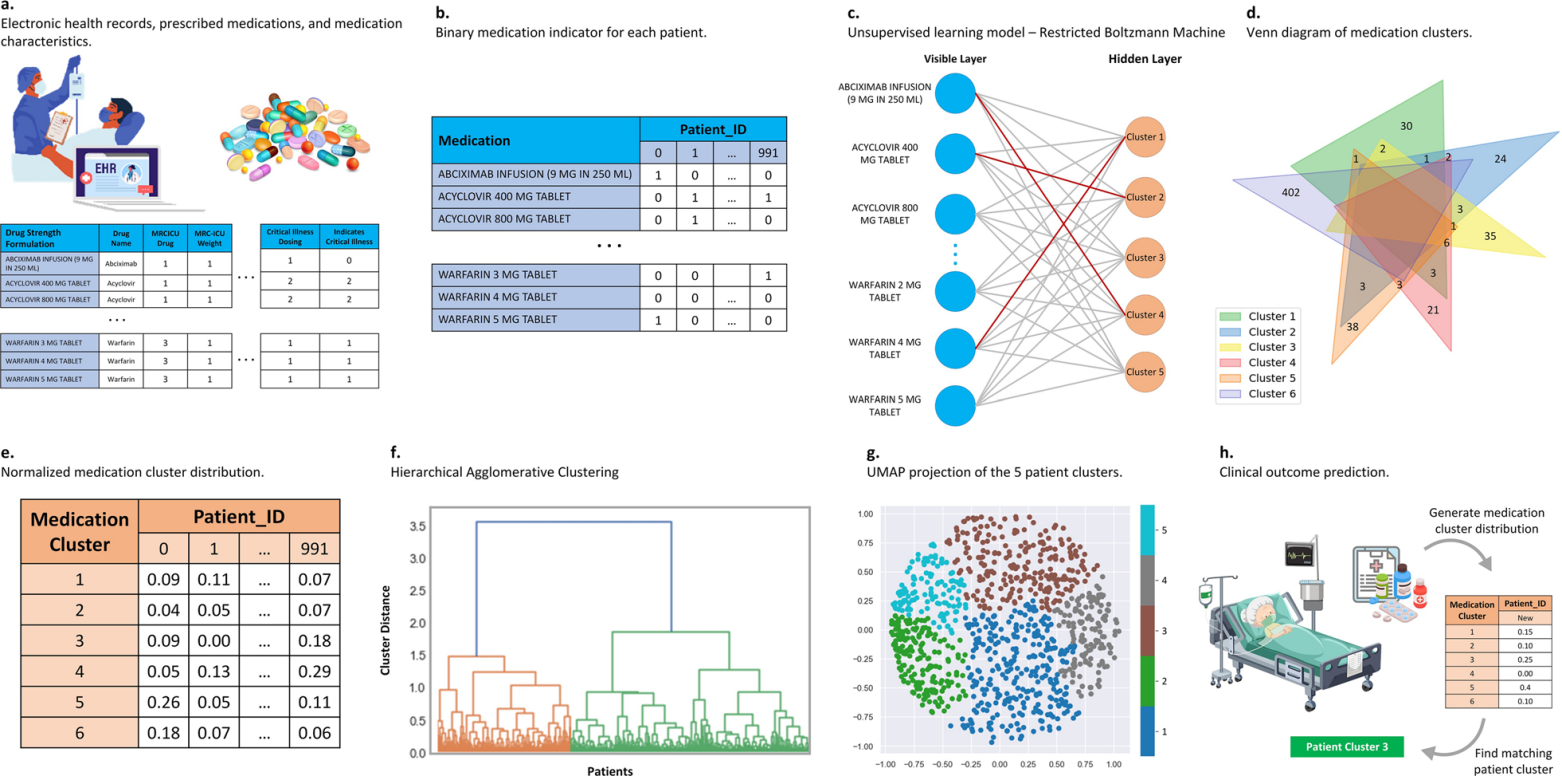
Pharmacophenotype derivation workflow. **a** Medication administration records (including drug name, dose, formulation, route, and time) as well as other relevant patient data are recorded in the electronic health record (EHR) system. **b** The MAR data was processed to indicate 0 for not receiving that medication and 1 for receiving that medication for each patient. **c** Using restricted Boltzmann machine, six pharmacophenotypes were generated. If the medication from the visible layer was not assigned to a hidden layer, that medication was grouped in the sixth or unassigned cluster. **d** The pharmacophenotypes are displayed in a Venn diagram describing the degree of overlap between the clusters and how the medications are distributed among the clusters. **e** The frequency of every pharmacophenotype is counted and normalized by considering the total medications taken by every patient during their stay. **f** The resulting normalized pharmacophenotype distribution of every patient was used as a feature in the agglomerative hierarchical clustering method to develop novel pharmacophenotypes of critically ill patients. **g** The Uniform Manifold Approximation and Projection (UMAP) for Dimension Reduction of the five patient clusters was performed. **h** These novel pharmacophenotypes were associated with unique patterns of patient outcomes. MRC-ICU – medication regimen complexity in the intensive care unit

Hierarchical agglomerative clustering is a bottom-up clustering approach in which each observation is initially considered a single cluster that was used for patient clustering [35]. Two clusters with the highest similarity then merge into a new bigger cluster, and this process is iterated until all observations are a member of one single cluster. To cluster patients using hierarchical agglomerative clustering, the normalized table of pharmacophenotypes from the previous step was used. The number of clusters (*n* = 5) was optimized through the visual inspection of the dendrogram, which illustrates the hierarchical relationship of the observations. Figure 1 summarizes the pharmacophenotype derivation workflow. Python 3.8.8 and scikit-learn 1.1.3 library were used for the implementation of all methods.

#### Similarity analysis of patient clusters

After performing patient clustering with the optimal number of clusters, we analyzed the clusters to reveal if the comparison of patient outcomes with medication data could distinguish clinically relevant characteristics. Two statistical tests were performed for different characteristics, Wilcoxon rank sum and signed rank tests for continuous characteristics and Fisher's exact tests for categorical characteristics. Holm's approach for adjustment of *p* values was also considered to control the familywise error rates for the comparisons within each outcome, and the significance level was assessed at *p* value < 0.05.

## Results

**Fig. 2:**
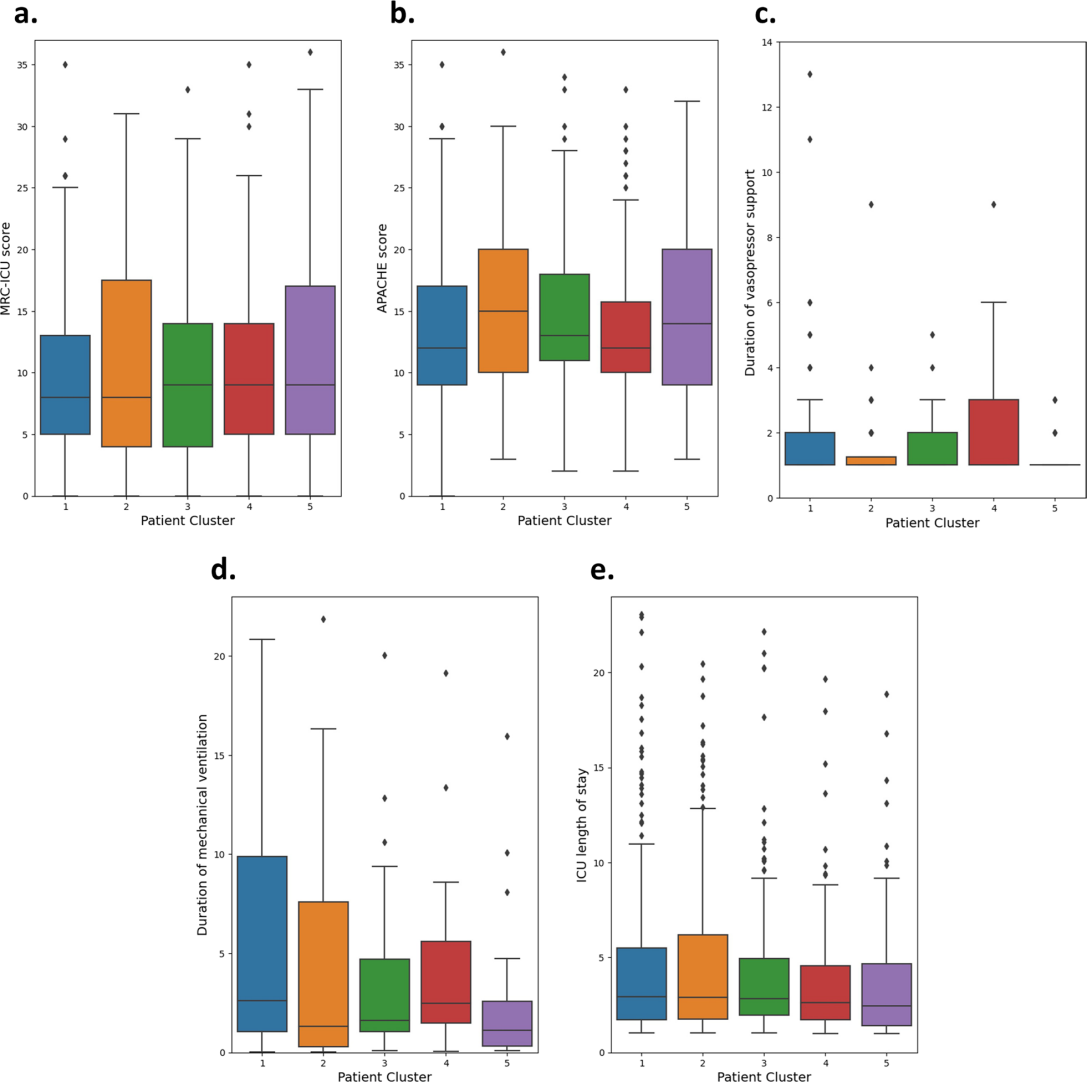
Boxplots of different patient outcomes for patient clusters*.* **a** MRC-ICU score evaluated 24 h after ICU admission. **b** APACHE II score evaluated 24 h after ICU admission. **c** Total days of vasopressor support. **d** Total days of mechanical ventilation. **e** Total days of ICU admission. For panels d and e, outlier records were omitted to improve the visibility of the distribution. ICU—intensive care unit; APACHE—Acute Physiology and Chronic Health Evaluation; MRC-ICU—medication regimen complexity in the ICU

**Fig. 3:**
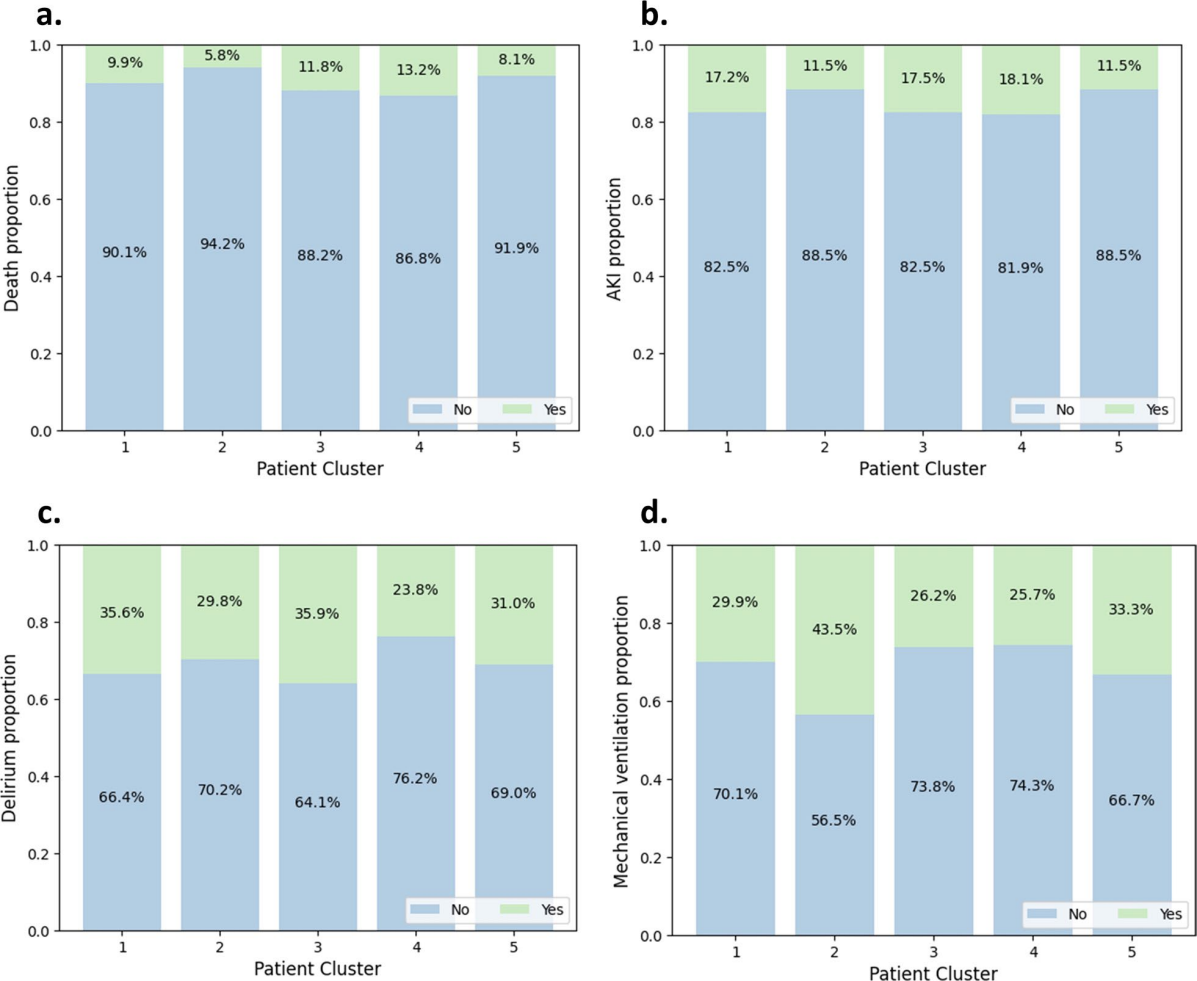
Stacked bar plots of different categorical patient outcomes for patient clusters. **a** Death proportion. **b** Acute kidney injury (AKI) presence proportion **c** Delirium presence proportion. **d** Mechanical ventilation presence proportion. Patients with unreported or unknown outcomes were omitted from the analysis

The demographic features of the 991 patients included in the study are summarized in Table 1. The average APACHE II score was 14.3 ± 6.4, average age was 61.2 ± 17.6, and average MRC-ICU score at 24 h was 10.3 ± 7.7. The medical ICU (40.8%) was the most common ICU setting, and a total of 9.8% (97) died in the ICU. Figure 2 compares the continuous outcomes between the five patient clusters, and Fig. 3 compares the dichotomous outcome between the five clusters.

**Table 1:** Demographic characteristics by patient cluster

Cluster (patient number)	1 (*N* = 304)	2 (*N* = 191)	3 (*N* = 229)	4 (*N* = 144)	5 (*N* = 123)	Total (*N* = 991)
Age (years)	59.7 ± 17.5	63.2 ± 16.5	61.4 ± 18.3	61.1 ± 18.1	61.9 ± 17.3	61.2 ± 17.6
Sex (female)	136 (44.7)	80 (41.9)	96 (41.9)	69 (47.9)	47 (38.2)	428 (43.2)
ICU type						
Medical	111 (36.5)	70 (36.6)	104 (45.4)	54 (37.5)	65 (52.8)	404 (40.8)
Cardiac	67 (22.0)	79 (41.4)	70 (34.5)	40 (27.8)	40 (32.5)	296 (29.9)
Surgical	27 (8.9)	19 (9.9)	29 (12.7)	13 (9.0)	9 (7.3)	97 (9.8)
Neurosciences	45 (14.8)	9 (4.7)	12 (5.2)	26 (18.1)	1 (0.8)	93 (9.4)
Burn	47 (15.5)	7 (3.7)	3 (1.3)	11 (7.6)	2 (1.6)	70 (7.1)
Other	7 (2.3)	7 (3.7)	11 (4.8)	0 (0.0)	6 (4.9)	31 (3.1)
Admission diagnosis						
Cardiovascular	63 (20.7)	71 (37.2)	47 (20.5)	32 (22.2)	31 (25.2)	244 (24.6)
Neurology	41 (13.5)	22 (11.5)	22 (9.6)	27 (18.8)	9 (7.3)	121 (12.2)
Pulmonary	22 (7.2)	15 (7.9)	27 (11.8)	10 (6.9)	14 (11.4)	88 (8.9)
Sepsis/infection	17 (5.6)	8 (4.2)	26 (11.4)	8 (5.6)	16 (13.0)	75 (7.6)
Gastrointestinal	16 (5.3)	12 (6.3)	25 (10.9)	9 (6.3)	10 (8.1)	72 (7.3)
Neoplasm	20 (6.6)	6 (3.1)	13 (5.7)	8 (5.6)	2 (1.6)	49 (4.9)
Trauma	15 (4.9)	10 (5.2)	14 (6.1)	6 (4.2)	4 (3.3)	49 (4.9)
Renal	5 (1.6)	2 (1)	9 (3.9)	6 (4.2)	3 (2.4)	25 (2.5)
Endocrine	8 (2.6)	4 (2.1)	5 (2.2)	4 (2.8)	3 (2.4)	24 (2.4)
Other	97 (31.9)	41 (21.5)	41 (17.9)	34 (23.6)	31 (25.2)	244 (24.6)
APACHE II at 24 h	13.5 ± 6.4	15.7 ± 6.4	14.6 ± 5.9	13.3 ± 5.9	14.7 ± 7.0	14.3 ± 6.4
MRC-ICU at 24 h	9.4 ± 6.5	11.6 ± 9.4	10 ± 7.2	10.1 ± 7.0	11.2 ± 8.3	10.3 ± 7.7
Mortality	30 (9.9)	11 (5.8)	27 (11.8)	19 (13.2)	10 (8.1)	97 (9.8)
Hospital length of stay (days)	12.3 ± 20.3	14.3 ± 30.5	11.4 ± 16.2	8.6 ± 8.8	8.3 ± 7.9	11.5 ± 19.7
ICU length of stay (days)	6.2 ± 12.1	5.6 ± 9.9	4.7 ± 9.1	4.3 ± 6.3	3.6 ± 3.2	6.3 ± 14.3
Presence of delirium *n* (%, total)	96 (35.6, 270)	54 (29.8, 181)	78 (35.9, 217)	30 (23.8, 126)	35 (31.0, 113)	293 (32.6, 907)
Acute kidney injury *n* (%, total)	52 (17.2, 303)	22 (11.5, 191)	40 (17.5, 229)	26 (18.1, 144)	14 (11.5, 122)	154 (15.6, 989)
Duration of vasopressors support (days)	2.1 ± 2.1	1.4 ± 1.1	1.5 ± 0.8	2.1 ± 1.7	1.2 ± 0.5	1.7 ± 1.4
Presence of mechanical ventilation	91 (29.9)	83 (43.5)	60 (26.2)	37 (25.7)	41 (33.3)	312 (31.5)
Duration of mechanical ventilation (days)	9.1 ± 19.3	5.1 ± 11.5	3.4 ± 4.6	5.8 ± 10.2	2.1 ± 3.1	5.6 ± 12.9
Presence of fluid overload (%, total)	47 (17.9, 262)	26 (15.0, 173)	37 (18.9, 197)	19 (15.3, 124)	23 (22.1, 104)	158 (18.4, 860)

Data are presented as *n* (%) or mean ± standard deviation (SD) unless otherwise stated

*ICU* intensive care unit, *APACHE *Acute Physiology and Chronic Health Evaluation, *MRC-ICU* medication regimen complexity in the ICU

In total, the patients received 30,550 medications during their first 24 h in the ICU. When drug name, dose, strength, and formulation (e.g., amoxicillin/clavulanate 875/125 mg tablet) were applied to this list, 543 different medications were used. Restricted Boltzmann machine assigned 152 medications to five pharmacophenotypes (Additional file 1: Table S1) with a sixth pharmacophenotype consisting of 391 unassigned medications (Additional file 1: Table S2). Table 2 provides a descriptive characterization of the six pharmacophenotypes identified. Medications in all six pharmacophenotypes were highly represented by presence in the MRC-ICU Score (range of 95.7% to 100%). The MRC-ICU Score incorporates a weighted system with drugs requiring more monitoring or oversight due to narrow therapeutic index having higher weights. More highly weighted drugs per the MRC-ICU score were present in Pharmacophenotype 2 with the lowest representation of higher weights in Pharmacophenotype 1. Generally, the medications given in the first 24 h of the ICU stay were associated with critical illness and/or an intravenous route and the use of a more invasive medication administration route (e.g., intravenous instead of oral).

**Table 2:** Pair-wise comparison of differences in patient outcomes by patient cluster

1st Cluster	2nd Cluster	Continuous outcomes					Categorical outcomes			
		Length of stay (days)	Duration of mechanical ventilation (days)	Duration of vasopressor support (days)	APACHE II score (first 24 h)	MRC-ICU	Death	Acute kidney injury	Delirium	Mechanical ventilation
1	2	0.9971	0.0027	0.0136	0.0003	0.1937	0.1316	0.0940	0.7607	0.9279
1	3	0.9760	0.0603	0.0896	0.0107	0.5721	0.4827	1.0000	0.3018	0.3824
1	4	0.1559	0.6876	0.9624	0.8643	0.4727	0.3311	0.3620	0.1300	0.3724
1	5	0.0258	0.0002	0.0011	0.1407	0.2410	0.7143	0.1823	1.0000	0.4903
2	3	0.8934	0.2221	0.3854	0.1563	0.3545	0.0397	0.1705	0.2018	0.3226
2	4	0.1686	0.0867	0.0488	0.0019	0.6489	0.2090	0.0225	0.2979	0.3633
2	5	0.0355	0.2300	0.2499	0.1904	0.9259	0.4891	1.0000	0.8963	0.5597
3	4	0.1278	0.1279	0.1613	0.0368	0.8586	0.7040	0.3835	0.0220	1.0000
3	5	0.0257	0.0185	0.0539	0.8206	0.4115	0.3628	0.2390	0.3939	0.1748
4	5	0.4197	0.0053	0.0077	0.1781	0.6249	0.2370	0.0472	0.2451	0.1797

This analysis summarizes the significant differences observed between two patient clusters (e.g., Cluster 1 and Cluster 2) for various outcomes

Table 3 summarizes the various pair-wise comparison of patient outcomes by each patient cluster. Patient Clusters 1, 2, and 3 had significantly different lengths of stay compared to Cluster 5 (with Cluster 5 having the lowest overall ICU length of stay). Cluster 5 also had the shortest duration of mechanical ventilation, which was significantly different compared to Clusters 1, 3, and 4. The MRC-ICU was not significantly different among any of the patient clusters. Mortality was lowest in Patient Cluster 2 despite patients in this cluster having the highest relative APACHE II and MRC-ICU scores in comparison and a longer duration of ICU stay.

**Table 3:** Descriptive characterization of pharmacophenotypes by medication class

	1	2	3	4	5	6
MRC-ICU drugs	36 (97.3)	33 (100.0)	45 (95.7)	35 (100.0)	44 (95.7)	391 (97.3)
Average weight value of MRC-ICU drugs	1.2 (0.6)	1.4 (0.7)	1.3 (0.7)	1.4 (0.7)	1.2 (0.6)	1.3 (0.7)
MRC-ICU weight of 1	29 (78.4)	22 (66.7)	32 (68.1)	25 (71.4)	33 (71.7)	288 (71.6)
MRC-ICU weight of 2	3 (8.11)	7 (21.2)	9 (19.1)	5 (14.3)	7 (15.2)	61 (15.2)
MRC-ICU weight of 3	3 (8.1)	3 (9.1)	4 (8.5)	5 (14.3)	2 (4.3)	36 (9.0)
Analgesic	5 (13.5)	4 (12.1)	6 (12.8)	4 (11.4)	4 (8.7)	49 (12.2)
Antiarrhythmics	1 (2.7)	2 (6.1)	2 (4.2)	1 (2.9)	0 (0)	18 (4.5)
Antibiotic	9 (24.3)	4 (12.1)	11 (23.4)	9 (25.7)	13 (28.3)	98 (24.3)
Anticoagulant	3 (8.1)	0 (0)	3 (6.4)	2 (5.7)	2 (4.3)	36 (8.9)
Anticonvulsants	0 (0)	0 (0)	1 (2.1)	3 (8.6)	1 (2.2)	8 (2.0)
Antidotes/rescue	2 (5.4)	0 (0)	0 (0)	0 (0)	0 (0)	4 (1.0)
Antifungal agents	0 (0)	1 (3.0)	1 (2.1)	1 (2.9)	2 (4.3)	7 (1.7)
Antihypertensive	2 (5.4)	2 (6.1)	0 (0)	1 (2.9)	2 (4.3)	7 (1.7)
Antiplatelet	0 (0)	0 (0)	0 (0)	0 (0)	1 (2.2)	1 (0.2)
Antiprotozoal agent	0 (0)	0 (0)	0 (0)	0 (0)	0 (0)	2 (0.5)
Antipsychotic	0 (0)	0 (0)	0 (0)	0 (0)	0 (0)	1 (0.2)
Antiviral agent	0 (0)	2 (6.1)	1 (2.1)	0 (0)	0 (0)	12 (3.0)
COPD/asthma	0 (0)	0 (0)	0 (0)	0 (0)	0 (0)	2 (0.5)
Chemotherapy	2 (5.4)	1 (3.0)	0 (0)	0 (0)	0 (0)	2 (0.5)
Diabetic agent	1 (2.7)	1 (3.0)	0 (0)	1 (2.9)	1 (2.2)	14 (3.5)
Diuretic	0 (0)	0 (0)	0 (0)	0 (0)	1 (2.2)	3 (0.7)
Factor product	0 (0)	0 (0)	1 (2.1)	0 (0)	0 (0)	0 (0)
Fluids	4 (10.8)	1 (3.0)	4 (8.5)	1 (2.9)	0 (0)	21 (5.2)
Gastrointestinal	0 (0)	2 (6.1)	3 (6.4)	1 (2.9)	4 (8.7)	14 (3.5)
Gastric agents	1 (2.7)	1 (3.0)	1 (2.1)	1 (2.9)	1 (2.2)	11 (2.7)
Hypertonic saline	0 (0)	0 (0)	0 (0)	0 (0)	0 (0)	4 (1.0)
Immunosuppressant	0 (0)	1 (3.0)	1 (2.1)	0 (0)	4 (8.7)	9 (2.2)
Intropic agent	0 (0)	1 (3.0)	0 (0)	0 (0)	0 (0)	0 (0)
Laxative	0 (0)	0 (0)	1 (2.1)	0 (0)	0 (0)	2 (0.5)
Neuromuscular blocking agent	0 (0)	2 (6.1)	2 (4.2)	0 (0)	0 (0)	11 (2.7)
Opioid reversal	0 (0)	0 (0)	1 (2.1)	1 (2.9)	0 (0)	1 (0.2)
Sedative agents	1 (2.7)	5 (15.1)	4 (8.5)	4 (11.4)	3 (6.5)	27 (6.7)
Somatostatic agents	1 (2.7)	0 (0)	0 (0)	0 (0)	0 (0)	4 (1.0)
TPN	0 (0)	0 (0)	0 (0)	0 (0)	1 (2.2)	3 (0.7)
Vasoactive agent	0 (0)	1 (3.0)	0 (0)	0 (0)	1 (2.2)	2 (0.5)
Vasodilator	0 (0)	0 (0)	0 (0)	0 (0)	0 (0)	1 (0.2)
Vasopressor	5 (13.5)	2 (6.1)	3 (6.4)	5 (14.3)	5 (10.9)	28 (6.9)
Drug indicates critical illness (always)	12 (32.4)	13 (39.4)	7 (14.9)	11 (31.4)	11 (23.9)	91 (22.6)
Drug indicates critical illness (maybe)	17 (45.9)	11 (33.3)	22 (46.8)	17 (48.6)	21 (45.7)	189 (47.0)
Intravenous route	27 (73.0)	22 (66.7)	29 (61.7)	25 (71.4)	30 (65.2)	253 (62.9)
Route escalation	20 (54.1)	17 (51.6)	20 (42.6)	23 (65.7)	12 (26.1)	182 (45.3)

Data are displayed as *n* (%) or mean (SD) unless otherwise stated

*MRC-ICU* medical regimen complexity in the intensive care unit, *COPD* chronic obstructive pulmonary disease, *TPN* total parenteral nutrition

**Fig. 4:**
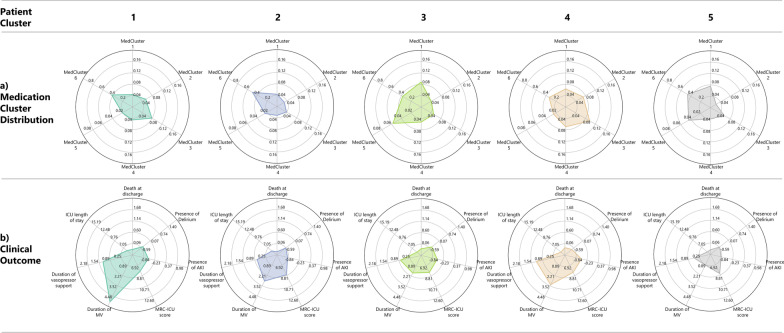
Radar charts of pharmacophenotypes and patient outcome distribution. **a** Radar chart of the mean pharmacophenotype distribution for different patient clusters. **b** Radar chart of the mean clinical outcomes for different patient clusters. The further the mean value toward the edge of each axis, the more severe the outcome. Thus, Patient Cluster 5 relatively has the least serious outcomes, while Patient Clusters 1 and 4 have more severe outcomes. AKI—acute kidney injury; ICU—intensive care unit; MRC-ICU—medication regimen complexity in the ICU; MV—mechanical ventilation

Despite similar severity of illness and medication regimen complexity (as measured by the APACHE II and MRC-ICU, respectively), patient outcomes varied among the pharmacophenotypes. These are depicted in the radar plot of Fig. 4, which organizes both pharmacophenotypes and patient outcomes by each patient cluster. Patient Cluster 4 has a well-rounded distribution of all pharmacophenotypes compared to other patient clusters. In contrast, Patient Cluster 2 has a notably high distribution in Pharmacophenotype 6. Patient Cluster 5 had about half the ICU length of stay of Patient Cluster 1: here, Patient Cluster 1 had nearly double the distributions of Pharmacophenotypes 1 and 3, and Patient Cluster 5 had more exposure to Pharmacophenotype 5. Patient Clusters 1 and 2 had similar distributions of Pharmacophenotype 6 but less of Pharmacophenotypes 2 and 4 comparatively, with a significantly lower duration of mechanical ventilation compared to other clusters.

## Discussion

In the first unsupervised machine learning analysis of the entire medication administration record in the first 24 h of an ICU stay, six pharmacophenotypes were identified that had varying distributions across five unique patient clusters. These patient clusters had significantly different patterns in terms of patient-centered outcomes and ICU-related complications. This study is the first to apply artificial intelligence to complete medication administration data enhanced with a ICU medication-specific common data model; these methods demonstrated the ability to categorize patients by outcomes and may serve as a foundation for the future use of artificial intelligence in the ICU.

Critically ill patients are known for their diagnostic and medical complexity, which directly results in the use of more complex medication regimens. Computating these complex, heterogenous ICU medication regimens that can guide clinical-decision making has notable parallels to the management of heterogenous syndromes like ARDS and sepsis [12–19]. The concept of phenotyping and using artificial intelligence methods to drive that phenotyping process has gained traction for its ability to parse this heterogeneity into more meaningful sub-groups (i.e., phenotypes) that appear to have unique patterns in treatment response [20–23]. Indeed, previously ‘negative’ ARDS trials have shown that one phenotype did have mortality benefit [20, 21]. Our methodology applied an unsupervised feature learning approach with restricted Boltzmann machine, in combination with a common data model, to identify six pharmacophenotypes. The distribution patterns among these pharmacophenotypes were different across the identified patient clusters. Notably, in this analysis of the first 24 h of the medication regimens, almost every medication in the pharmacophenotypes identified is captured by the MRC-ICU Scoring Tool, a clinician designed tool intended to capture complex, high-risk medications that require specialized monitoring and oversight for safe and appropriate use [1, 7–9, 36]. Interestingly, Patient Cluster 2 had the lowest mortality despite the highest relative severity of illness and highest MRC-ICU score. Though causal inference is limited by the present study design, a potential hypothesis is that within the extremes of critical illness (i.e., high APACHE II scores), there are patients that are highly likely to benefit from ‘complex’ medication therapy (e.g., multi-drug-resistant septic shock requiring multiple broad-spectrum antibiotics and combination vasoactive drug therapy) while another category of patients either requires non-medication therapy (e.g., surgical intervention) or is approaching of end-of-life that is beyond the scope of available interventions. As such, within the high APACHE II score strata, there are patients more and less likely to benefit from high intensity medication therapy. Though these examples are potentially visible to the clinician eye without the need for artificial intelligence based alerts, subtleties of presentation captured by such a tool may guide a clinician’s decision-making.

The novel methodologies in the present study show early promise in the ability to cluster illness severity (e.g., APACHE II) with required ICU interventions (e.g., mechanical ventilation, complex medication regimens), and outcomes (e.g., mortality). Though beyond the scope of this analysis, these findings may serve as a foundation for prediction of ICU complications that could be prevented with timely intervention by incorporating medication-related data. While existing software to improve medication safety has improved tremendously with regard to dose checking and drug-drug interaction identification, nuanced analysis of risks and benefits of ‘reasonable’ drug combinations remains out of the range of present day software. For example, the use of hydromorphone and midazolam within commonly prescribed dosing ranges would not be captured as a ‘medication error’ and is often a clinically reasonable combination; however, in certain situations based on patient-specific factors, this combination may result in a unpalatably high risk of oversedation and need for emergent intubation that causes the clinician to consider alternative agents. Thus, the future of this type of analysis may be alerts based on potential risks that support clinical decision-making as it relates to risk–benefit of each medication-related decision.

Artificial intelligence requires a validated common data model to improve outcomes in critically ill patients, and thus, a major goal of the data science community has been to harmonize and standardize the substantial amount of data in the EHR [37, 38]. Machine-readable, standardized common data models facilitate reproducibility and generalizability across datasets, so without their incorporation in AI efforts, the reproducibility and external validity of these efforts may be limited [39]. The importance of common data model development and use was internationally-recognized with the publication of the FAIR Guiding Principles, which are intended to steward patient data to be Findable, Accessible, Interoperable, and Reusable [40]. Through efforts such as the Observational Medical Outcomes Partnership (OMOP) Common Data Model and RxNorm, application of these principles to vast amounts of data generated by ICU patients has begun, though ICU medication data has remained largely untouched, which is a gap given the important relationship between ICU medications and ICU outcomes [25, 39, 41, 42]. When optimizing ICU medication management to improve outcomes, clinicians apply nuanced ICU medication knowledge to balance medication benefits with known risks on a patient-specific basis [43]. Key medication features in this complex clinical decision-making process include synergistic mechanisms of action, additive adverse drug event risk (side effect profiles often overlap), and the effects of critical illness on pharmacokinetic parameters and pharmacodynamic response [43]. Thus, existing drug terminology focused only on standardizing drug products across databases are limited by the degree of contextualization that they may provide to learning algorithms. Artificial intelligence can improve patient-centered outcomes by predicting adverse drug event risks and identifying optimal medication interventions [44]; however, current common data models that support artificial intelligence include only basic features (e.g., drug, dose, route) and fail to capture many clinically relevant medications features necessary for clinician decision-making [24, 45]. As such, this analysis marks a significant first step in the exploration of the application of common data models incorporating clinical features and appropriate contextualization to the ICU medication space.

Our study has several limitations. The present approach included a diverse range of ICUs (with their associated admission diagnoses); however, medication regimens are generally tailored to individual disease states. As such, the possibility exists that the granularity of evaluating pharmacophenotypes in specific disease states (e.g., ARDS) may reveal more distinct patterns. Future analyses may benefit from Charlson Comorbidity Index or other comorbidity inclusion, as these have the potential to influence medication therapy. Despite our assumption of homogeneity across medication regimens, and the use of a clustering approach with limited expressiveness, such as restricted Boltzmann machine, the reality may be that these relationships are highly intricate and have a noisy interplay. The learned representations in restricted Boltzmann machines are often difficult to interpret, which can make it challenging to gain insights into the underlying structure of the data [46]. Therefore, we seek to use variational autoencoders in future studies to increase the ability to capture more complex patterns and interoperability of the workflow. Moreover, expanding future analysis to include timepoints beyond for medication therapy beyond 24 h is warranted. Finally, the observational nature of this study precludes causal inference between medications and outcomes, and future analysis will be required to biologically link these observations in a way that reduces the ‘black box effect’ of artificial intelligence for the end-user. Even with these limitations, this analysis marks the first time the complete medication profile interpreted via a common data model has been conducted for ICU patient outcomes.

## Conclusion

The complexity of medication regimens for critically ill patients may be better understood by the application of pharmacophenotypes. Further exploration of the intertwined relationship among disease, medication treatment, medical intervention, and patient-centered outcomes, the use of unsupervised learning methods, particularly via the support of common data models, warrants further investigation.

## Supplementary Information

**Additional file 1. Table 1.** Pharmacophenotypes Assigned by Restricted Boltzmann Machine. **Table 2.** Pharmacophenotype 6 - Medications unassigned through Restricted Boltzmann Machine.

## Data Availability

Datasets may be provided upon request.
