# Supplementary material for: Pharmacophenotype identification of intensive care unit medications using unsupervised cluster analysis of the ICURx common data model
Source: Crit Care. 2023 May 2;27:167. doi: 10.1186/s13054-023-04437-2 (PMC10155304; doi:10.1186/s13054-023-04437-2)
Supplement: Supplementary file 1 — Additional file 1. Table 1. Pharmacophenotypes Assigned by Restricted Boltzmann Machine. Table 2. Pharmacophenotype 6 - Medications unassigned through Restricted Boltzmann Machine. [file 13054_2023_4437_MOESM1_ESM.docx]

**Supplementary Material**

**Table 1. Pharmacophenotypes Assigned by Restricted Boltzmann Machine**

| **Pharmacophenotype 1** | **Pharmacophenotype 2** | **Pharmacophenotype 3** | **Pharmacophenotype 4** | **Pharmacophenotype 5** |
| --- | --- | --- | --- | --- |
| Alteplase 0.09 mg/kg stroke bolus  Amiodarone infusion 450 mg/250 ml (1.8 mg/ml) in d5w  Amoxicillin/clavulanate 875/125 mg tablet  Bicalutamide 50 mg tablet  Cefazolin 1 g/50 ml in dextrose 4% (50 ml)  Ceftriaxone 1 gram/50 ml in dextrose (iso-osmot) intravenous piggyback  Doxycycline hyclate 100 mg tablet/capsule wrapper  Ertapenem (invanz) ivpb (adult)  Fentanyl (pf) 10 mcg/ml in 0.9 % sodium chloride intravenous superorderable  Fentanyl 12 mcg/hr transdermal patch  Heparin (porcine) 10,000 unit/1,000 ml in ns (unch cupid)  Heparin, porcine (pf) 100 unit/ml injection  Heparin, porcine (pf) 5,000 unit/0.5 ml injection syringe  Hydromorphone 2 mg tablet  Insulin glargine (u-300) conc. 300 unit/ml (1.5 ml) subcutaneous pen  Ketamine (ketalar) 50 mg/ml injection  Lactated ringers iv bolus  Morphine 1 mg/ml in 0.9 % sodium chloride injectable pump reservoir  Nicardipine in nacl 40 mg/200 ml infusion  Nintedanib 150 mg capsule  Nitroprusside 50 mg/100 ml (500 mcg/ml) in d5w infusion  Norepinephrine infusion in 250 ml  Octreotide 500 mcg/100 ml (5 mcg/ml) in ns infusion  Oxycodone 10 mg tablet  Pantoprazole 40 mg intravenous solution  Phenylephrine in 0.9% nacl(pf) 20 mg/250 ml (80 mcg/ml) infusion  Phenylephrine infusion 100 mg/250 ml (400 mcg/ml) in ns  Piperacillin-tazobactam 3.375 gram/50 ml dextrose(iso-os) iv piggyback  Prothrombin complex (kcentra) intermittent infusion  Sodium acetate (4meq/ml) in 1000 ml continuous infusion  Sodium bicarbonate drip  Sodium chloride 0.9 % intravenous solution  Vancomycin 10 mg/ml max concentrated ivpb  Vancomycin 1250 mg in 250 ml ivpb  Vancomycin ivpb in 100 ml  Vasopressin (pitressin) infusion 50 units/50 ml  Vasopressin 40 units/50 ml (0.8 unit/ml) in ns infusion | Acyclovir ivpb in 250 ml  Amiodarone 200 mg tablet  Azithromycin ivpb in 250 ml  Aztreonam ivpb 1 gram mini-bag plus (components)  Bisacodyl 5 mg tablet,delayed release  Cisatracurium (adult) infusion  Digoxin 250 mcg tablet  Dobutamine (dobutrex) 1,000 mg 1,000 mg/250 ml (4,000 mcg/ml) in dextrose 5% 250 ml (4000 mcg/ml) infusion  Emtricitabine-tenofovir 200 mg-300 mg tablet  Famotidine (pf) (pepcid) 20 mg/2 ml injection  Fentanyl (pf) (sublimaze) 50 mcg/ml injection  Fentanyl 10 mcg/ml in d5 or ns continuous infusion 150ml  Fluconazole 10 mg/ml oral suspension  Insulin regular (humulin,novolin) 100 unit/ml injection  Isoproterenol infusion 4 mcg/ml  Labetalol infusion 300 mg/60 ml (5 mg/ml)  Lactated ringers iv bolus  Meropenem in 100 ml ivpb  Midazolam (pf) 1 mg/ml in 0.9 % sodium chloride intravenous solution  Midazolam 100 mg/100 ml (1 mg/ml) in ns/d5w infn  Midazolam 5 mg/ml (combined) injection solution  Midazolam infusion  Milrinone 40 mg in 5% dextrose 200 ml (200 mcg/ml) 40 mg/200 ml (200 mcg/ml) infusion  Mirtazapine 15 mg disintegrating tablet  Morphine (pf) 1 mg/ml in 0.9% sodium chloride intravenous solution  Morphine er 30 mg tablet,extended release  Nintedanib 150 mg capsule  Nitroglycerin 100 mg/250 ml (400 mcg/ml) in 5 % dextrose intravenous  Phenylephrine 100mg in 250ml ns infusion  Smog enema 240ml  Tacrolimus 1 mg/ml oral suspension  Vancomycin 1000 mg in 250 ml ivpb vialmate  Vecuronium (norcuron) 10 mg injection | Amoxicillin 500 mg-potassium clavulanate 125 mg tablet  Ampicillin-sulbactam ivpb 1.5 gram mini-bag plus  Azithromycin ivpb in 250 ml  Cefazolin 2 g/50 ml in dextrose 4% (50 ml)  Cefepime (maxipime) 1 g/100 ml connector bag  Cefepime (maxipime) 2 g/100 ml connector bag  Ceftriaxone 1 gram/50 ml in dextrose (iso-osmot) intravenous piggyback  Cefuroxime sodium 1.5 gram solution for injection  Cisatracurium (nimbex) 2 mg/ml injection  Dexmedetomidine 20 mcg/5 ml (bolus from bag)  Dextrose 5 % and 0.45 % nacl iv bolus  Diazepam 5 mg tablet  Digoxin 125 mcg tablet  Diltiazem 100 mg/100ml (1mg/ml) in 0.9% sod chloride infusion add-vantage  Divalproex 500 mg tablet,delayed release  Dobutamine 500 mg/250 ml (2,000 mcg/ml) in 5 % dextrose iv  Docusate sodium 100 mg capsule  Doxycycline hyclate 100 mg tablet/capsule  Factor viia recomb (novoseven) intermittent infusion  Fentanyl (pf) (sublimaze) 50 mcg/ml injection  Fentanyl 12 mcg/hr transdermal patch  Fondaparinux 2.5 mg/0.5 ml subcutaneous solution for injection  Heparin (porcine) 100 unit/ml bolus from infusion  Heparin (porcine) for crrt 25,000 unit/250 ml in 0.45 % sodium chloride iv soln  Hydromorphone 0.2mg/ml pca cadd  Ketamine (ketalar) 100 mg/ml injection  Lactated ringers intravenous solution  Lactated ringers irrigation solution  Lithium carbonate er 300 mg tablet,extended release  Lopinavir-ritonavir 400 mg-100 mg/5 ml oral solution  Lorazepam (ativan) 2 mg/ml injection  Magnesium citrate 1.745g/30 ml (296 ml) oral solution  Meropenem ivpb 500 mg connector bag  Midodrine 5 mg tablet  Morphine (pf) 1 mg/ml in 0.9% sodium chloride intravenous solution  Morphine 10 mg/ml injection solution  Mycophenolate mofetil 250 mg capsule  Naloxone infusion 16 mcg/ml in 250 ml  Oxycodone-acetaminophen 10 mg-325 mg tablet  Pantoprazole 2 mg/ml oral suspension  Peg-electrolyte solution 420 gram oral solution  Phenylephrine 20 mg/250 ml (80 mcg/ml) in 0.9% sodium chloride  Polyethylene glycol 3350 17 gram/dose oral powder  Posaconazole 100 mg tablet,delayed release  Sodium chloride 0.9 % iv bolus  Vancomycin 1.5 gram/500 ml in 0.9 % sodium chloride intravenous soln  Vecuronium 1 mg/ml iv infusion (unc only) | Amphotericin b liposomal (ambisome) ivpb in 190 ml d5w  Aztreonam ivpb 2 gram mini-bag plus  Cefazolin 2 gram/50 ml in dextrose superorderable  Daptomycin (cubicin) ivpb in 100 ml  Digoxin 250 mcg tablet  Divalproex 125 mg capsule,delayed release sprinkle  Doxycycline hyclate 100 mg tablet  Fentanyl (pf) 10 mcg/ml in 0.9 % sodium chloride intravenous superorderable  Fosphenytoin iv 100 ml infusion  Heparin (porcine) 25,000 unit/250 ml in 0.45 % sodium chloride iv soln  Heparin, porcine (pf) 5,000 unit/0.5 ml injection superorderable  Insulin glargine (u-100) 100 unit/ml subcutaneous solution  Metronidazole 50 mg/ml oral susp  Midazolam (pf) (versed) 1 mg/ml injection  Midazolam (pf) (versed) 5 mg/ml injection  Midazolam (pf) 1 mg/ml in 0.9 % sodium chloride intravenous solution  Midazolam (versed) 1 mg/ml injection  Morphine 10 mg/5 ml oral solution  Morphine 10 mg/ml injection syringe  Naloxone infusion 16 mcg/ml in 250 ml  Nitroglycerin 100 mg in dextrose 5% infusion 250 ml (400 mcg/ml) 100 mg/250 ml (400 mcg/ml) infusion  Norepinephrine bitartrate 8 mg/250 ml (32 mcg/ml) in 0.9 % nacl iv  Oxycodone-acetaminophen 10 mg-325 mg tablet  Pantoprazole 2 mg/ml oral suspension  Pantoprazole 80mg in 100ml ns infusion  Pentobarbital (nembutal) 50 mg/ml injection  Phenylephrine 20 mg/250 ml (80 mcg/ml) in 0.9 % sodium chloride iv  Phenylephrine 20 mg/250 ml (80 mcg/ml) in 0.9% sodium chloride  Piperacillin-tazobactam 3.375 gram/50 ml dextrose(iso-os) iv piggyback  Sodium chloride 0.9 % iv bolus  Vancomycin 1.5 gram/500 ml in 0.9 % sodium chloride intravenous soln  Vancomycin 25 mg/ml oral suspension  Vancomycin 750 mg in 250 ml ivpb vialmate  Vasopressin (pitpressin) 20 units in ns 50 ml  Vasopressin 40 units/50 ml (0.8 unit/ml) in ns infusion | Acyclovir ivpb in 250 ml  Adult tpn rex  Alprazolam 1 mg tablet  Amoxicillin 400 mg-potassium clavulanate 57 mg/5 ml oral suspension  Amoxicillin/clavulanate 400/57 mg per 5 ml suspension (50 ml)  Amoxicillin/clavulanate 875/125 mg tablet  Basiliximab infusion  Bisacodyl 5 mg tablet,delayed release  Ceftaroline ivpb in 250 ml  Ceftriaxone 1 gram solution for injection  Ciprofloxacin 250 mg tablet  Clindamycin (cleocin) 900 mg/50 ml ivpb  Daptomycin (cubicin) ivpb in 100 ml  Dexmedetomidine 200 mcg/50 ml (4 mcg/ml) in dextrose 5% syringe (unc ped)  Dopamine 800 mg in dextose 5% 500 ml 800 mg/500 ml (1,600 mcg/ml) infusion  Dopamine 800 mg/500 ml (1,600 mcg/ml) in 5 % dextrose intravenous soln  Emtricitabine-tenofovir 200 mg-300 mg tablet  Enoxaparin 60 mg/0.6 ml subcutaneous syringe  Eptifibatide 0.75 mg/ml intravenous solution  Gentamicin ivpb in 100 ml (standard dosing)  Heparin (porcine) for crrt 25,000 unit/250 ml in 0.45 % sodium chloride iv soln  Hydrocodone 10 mg-chlorpheniramine 8 mg/5 ml oral susp extend.rel 12hr  Hydromorphone (pf) (dilaudid) 1 mg/ml injection  Insulin regular 1 unit/ml infusion  Isoproterenol infusion 4 mcg/ml  Lactulose 20 gram/30 ml oral solution  Lorazepam 2 mg tablet  Mannitol 20 % infusion  Morphine 2 mg/ml injection syringe  Morphine 4 mg/ml injection  Mycophenolate (cellcept) > 500 mg ivpb  Mycophenolate sodium 180 mg tablet,delayed release  Nicardipine (cardene) 20 mg in ns 200 mlآ  (0.1 mg/ml) 20 mg/200 ml infusion  Nitroglycerin 100 mg in dextrose 5% infusion 250 ml (400 mcg/ml) 100 mg/250 ml (400 mcg/ml) infusion  Norepinephrine bitartrate 8 mg/250 ml (32 mcg/ml) in dextrose 5 % iv  Oxacillin ivpb 2 g mini-bag plus  Pantoprazole 20 mg tablet,delayed release  Phenobarbital sodium 65 mg/ml injection solution  Phenylephrine 100mg in 250ml ns infusion  Piperacillin-tazobactam 3.375 g 100ml connector bag (extended infusion)  Piperacillin-tazobactam 3.375 gm/50ml dextrose (extended duration)  Senna 8.6 mg tablet  Sennosides 8.6 mg tablet  Tacrolimus 5 mg capsule  Vancomycin 1000 mg in 250 ml ivpb  Vasopressin (pitpressin) 20 units in ns 50 ml |

**Table 2. Pharmacophenotype 6 - Medications unassigned through Restricted Boltzmann Machine**

| Abciximab infusion (9 mg in 250 ml) - unc  Acyclovir 400 mg tablet  Acyclovir 800 mg tablet  Acyclovir ivpb in 100 ml  Adult 2-in-1 tpn unc template  Adult 3-in-1 tpn unc template  Albumin human 25 % bottle  Albumin human 5 % bottle  Albumin, human 5 % intravenous solution  Albumin, human 5 % intravenous solution  Albumin, human 5 % intravenous solution  Alprazolam 0.5 mg tablet  Alteplase (activase) 10 mg in 1000 ml ns (radiology)  Alteplase (activase) 100 mg injection  Alteplase 0.81 mg/kg stroke infusion  Amiodarone (cordarone) 50 mg/ml injection  Amiodarone 150 mg/100 ml (1.5 mg/ml) in dextrose, iso-osmotic iv  Amiodarone 360 mg/200 ml (1.8 mg/ml) in dextrose, iso-osmotic iv  Amiodarone 400 mg tablet  Amiodarone 450 mg/250 ml (1.8 mg/ml) in dextrose 5 % intravenous soln  Amiodarone infusion 750mg/500ml  Amiodarone infusion 900mg/500ml  Amiodarone iv bolus (adult)  Amoxicillin 400 mg-potassium clavulanate 57 mg/5 ml oral suspension  Amoxicillin 500 mg capsule  Amoxicillin 500 mg capsule  Amoxicillin 500 mg-potassium clavulanate 125 mg tablet  Amoxicillin 875 mg-potassium clavulanate 125 mg tablet  Amoxicillin 875 mg-potassium clavulanate 125 mg tablet  Amoxicillin/clavulanate 500/125 mg tablet  Ampicillin 2 gram connector bag  Ampicillin 2 gram connector bag  Ampicillin 2 gram iv solution  Ampicillin 2 gram mini-bag plus  Ampicillin 2 gram mini-bag plus  Ampicillin ivpb 1 gram 100 ml mini-bag plus  Ampicillin ivpb 1 gram 100 ml mini-bag plus  Ampicillin-sulbactam ivpb 3 gram connector bag  Apixaban 2.5 mg tablet  Apixaban 2.5 mg tablet  Apixaban 5 mg tablet  Argatroban 1 mg/ml in 0.9 % sodium chloride intravenous wrapper  Argatroban for crrt 50 mg/50 ml (1 mg/ml) in sodium chloride (iso-osmotic) iv  Azathioprine 50 mg tablet  Azithromycin ivpb in 190 ml  Aztreonam 1 gram solution for iv push  Aztreonam 2 gram solution for iv push  Aztreonam ivpb 1 gram connector bag  Belladonna alkaloids-opium 16.2 mg-30 mg rectal suppository  Belladonna alkaloids-opium 16.2 mg-60 mg rectal suppository  Bisacodyl 10 mg rectal suppository  Bivalirudin (angiomax) 250 mg injection  Bivalirudin 250 mg/50 ml bolus (cardiology) | Bivalirudin 250 mg/50 ml infusion (cardiology)  Cefazolin (ancef) 1 gram injection  Cefazolin 1 g iv solution pf  Cefazolin 1 gram/50 ml in dextrose (iso-osmotic) intravenous piggyback  Cefazolin 2 gram/100 ml in dextrose(iso-osmotic) intravenous piggyback  Cefazolin 2 gram/50 ml in dextrose (iso-osmotic) intravenous piggyback  Cefazolin 2 gram/50 ml in dextrose wrapper  Cefazolin in dextrose (iso-os) 2 gram/100 ml ivpb  Cefepime (maxipime) 1 g/100 ml connector bag  Cefepime (maxipime) 1gm/100ml extended infusion  Cefepime (maxipime) 2gm/100ml extended infusion  Cefepime 1 gram solution for injection  Cefepime 2 gram solution for iv push'  Ceftazidime (fortaz) 1 g mini-bag plus  Ceftazidime (fortaz) 2 g mini-bag plus  Ceftazidime ivpb in 100 ml  Ceftriaxone 2 gram/50 ml in dextrose (iso-osm) intravenous piggyback  Ceftriaxone ivpb 1 gram connector bag  Ceftriaxone ivpb 2 gram connector bag  Ceftriaxone ivpb in 100 ml  Cefuroxime (zinacef) 1.5 gram mini-bag plus  Cefuroxime (zinacef) ivpb doses <=750 mg/50 ml (adult  Cephalexin 500 mg capsule  Chlordiazepoxide 25 mg capsule  Chlorhexidine 0.12% oral rinse  Ciprofloxacin 100 mg tablet  Ciprofloxacin 500 mg tablet  Cisatracurium (nimbex) 1 mg/ml pediatric bolus from infusion  Cisatracurium infusion 1 mg/ml  Clevidipine 25 mg/50 ml intravenous emulsion  Clindamycin (cleocin) 600 mg/50 ml ivpb  Clindamycin 600 mg/50 ml in 5 % dextrose intravenous piggyback  Clindamycin 900 mg/50 ml in 5 % dextrose intravenous piggyback  Clonazepam 0.5 mg tablet  Clonazepam 1 mg tablet  Codeine 10 mg-guaifenesin 100 mg/5 ml oral liquid  Cyclosporine 1 mg/ml ns aviva iv  Cyclosporine 25 mg capsule  Cyclosporine modified 100 mg/ml oral solution  Daptomycin (cubicin) ivpb in 50 ml  Dexmedetomidine (precedex) (adult) infusion 100 ml  Dexmedetomidine (precedex) 200 mcg/5 ml syringe  Dexmedetomidine 200 mcg/50 ml (4 mcg/ml) in 0.9 % sodium chloride iv  Dexmedetomidine 400 mcg/100 ml (4 mcg/ml) in 0.9 % sodium chloride iv  Dextrose 10 % in water (d10w) intravenous solution  Dextrose 10 % iv bolus  Dextrose 10 % iv bolus | Dextrose 5 % and 0.45 % sodium chloride intravenous solution  Dextrose 5 % and 0.45 % sodium chloride intravenous solution  Dextrose 5 % and 0.9 % sodium chloride intravenous solution  Dextrose 5 % and lactated ringers intravenous solution  Dextrose 5 % in water (d5w) intravenous solution  Dopamine 400 mg/250 ml (1,600 mcg/ml) in 5 % dextrose intravenous soln  Dopamine 400 mg/250 ml (1,600 mcg/ml) in 5 % dextrose intravenous soln  Dopamine 6400 mcg/ml (adult max conc) in 250ml  Dopamine 6400 mcg/ml (adult max conc) in 250ml  Dopamine 800 mg/500 ml (1,600 mcg/ml) in 5 % dextrose intravenous soln  Doxycycline (vibramycin) ivpb (adult)  Doxycycline 100 mg mini-bag plus  Doxycycline 100 mg mini-bag plus  Doxycycline 100 mg/100 ml connector bag  Doxycycline 100 mg/100 ml connector bag  Doxycycline monohydrate 100 mg tablet  Enoxaparin 100 mg/ml subcutaneous syringe  Enoxaparin 120 mg/0.8 ml subcutaneous syringe  Enoxaparin 150 mg/ml subcutaneous syringe  Enoxaparin 30 mg/0.3 ml subcutaneous syringe  Enoxaparin 40 mg/0.4 ml subcutaneous syringe  Enoxaparin 80 mg/0.8 ml subcutaneous syringe  Epinephrine hcl 8 mg/250 ml (32 mcg/ml) in 5 % dextrose intravenous  Epinephrine hcl 8 mg/250 ml (32 mcg/ml) in 5 % dextrose intravenous  Epinephrine hcl in 5% dextrose 8 mg/250 ml (32 mcg/ml) infusion  Epinephrine hcl in d5w 8 mg/250 ml (32 mcg/ml) infusion  Epinephrine infusion 8 mg/250 ml (0.032 mg/ml) in d5w  Epinephrine infusion in 250 ml  Epinephrine infusion in 250 ml  Eptifibatide 2 mg/ml intravenous solution  Ertapenem (invanz) 1 gram injection  Ertapenem ivpb 1g 100ml mini-bag plus  Esmolol (brevibloc) 100 mg/10 ml (10 mg/ml) injection  Esmolol (brevibloc) 2,500 mg/250 ml (10 mg/ml) in sodium chloride 0.9% infusion 2,500 mg/250 ml (10 mg/ml) infusion  Esomeprazole magnesium dr 40 mg granules delayed release for susp  Etomidate (amidate) 2 mg/ml injection  Famotidine (pf) 20 mg/50 ml in 0.9 % nacl (iso) intravenous piggyback  Famotidine 10 mg/ml inj solution (multi-vial size)  Famotidine 20 mg tablet  Famotidine 40 mg tablet  Famotidine 40 mg/5 ml (8 mg/ml) oral suspension  Fat emulsion 20 % intravenous  Fentanyl (pf) 10 mcg/ml in 0.9 % sodium chloride intravenous |
| --- | --- | --- |
| Fentanyl (pf) 2,500 mcg/50 ml (50 mcg/ml) intravenous pca syringe  Fentanyl (pf) 50 mcg/ml intravenous solution  Fentanyl (sublimaze) 100 mcg in ns 50ml (rex or)  Fentanyl 100 mcg/hr transdermal patch  Fentanyl 10mcg/ml in ns continuous infusion 150ml  Fentanyl 25 mcg/hr transdermal patch  Fentanyl 50 mcg/hr transdermal patch  Flecainide 100 mg tablet  Fluconazole 100 mg tablet  Fluconazole 200 mg/100 ml in sod. chloride (iso) intravenous piggyback  Fluconazole 400 mg/200 ml in sod. chloride(iso) intravenous piggyback  Fosfomycin tromethamine 3 gram oral packet  Fosphenytoin iv 50 ml infusion  Furosemide infusion 2 mg/ml  Furosemide infusion 2 mg/ml  Ganciclovir (cytovene) ivpb  Glycerin (adult) rectal suppository  Guar gum oral packet  Heparin (porcine) (pf) 1,000 unit/500 ml in 0.9 % sodium chloride iv  Heparin (porcine) 1,000 unit/500 ml in 0.9% sodium chloride iv (combined)  Heparin (porcine) 1,000 unit/ml 1000 unit/ml injection  Heparin (porcine) 10,000 unit/1,000 ml in 0.9 % sod. chloride iv soln  Heparin (porcine) 10,000 unit/ml injection solution  Heparin (porcine) 100 unit/ml load from infusion  Heparin (porcine) 5,000 unit/ml (1 ml) injection cartridge  Heparin (porcine) 5,000 unit/ml inj solution (multi-vial size wrapper)  Heparin (porcine) 5,000 unit/ml injection solution  Heparin 10,000 units in ns 1000 ml  Heparin 30,000 units (cell saver) in 1000 ml ns  Heparin 7,500 units/0.75 ml syringe  Heparin, porcine (pf) 10 unit/ml injection  Hydrocodone 10 mg-acetaminophen 300 mg/15 ml oral solution  Hydrocodone 10 mg-acetaminophen 325 mg tablet  Hydrocodone 5 mg-acetaminophen 325 mg tablet  Hydrocodone-homatropine 5 mg-1.5 mg/5 ml (5 ml) oral syrup  Hydromorphone (dilaudid) 2 mg tablet  Hydromorphone (pf) (dilaudid) 1 mg/ml injection  Hydromorphone (pf) (dilaudid) 1 mg/ml injection  Hydromorphone 1 mg/ml (1 ml) in 0.9 % sodum chloride iv syringe  Hydromorphone 1 mg/ml in ns infusion wrapper  Hydromorphone 2 mg/ml injection solution  Hydromorphone 2 mg/ml injection syringe  Hydromorphone 4 mg/ml injection syringe  Hydromorphone 50 mg/50 ml (1 mg/ml) in 0.9 % sod.chloride iv pump resv  Hydromorphone infusion | Hydroxyurea 500 mg capsule  Insulin aspart (u-100) 100 unit/ml (3 ml) subcutaneous pen  Insulin aspart 100 unit/ml subcutaneous solution  Insulin degludec (u-100) 100 unit/ml (3 ml) subcutaneous pen  Insulin detemir 100 unit/ml subcutaneous solution  Insulin glargine (u-100) 100 unit/ml (3 ml) subcutaneous pen  Insulin human u-100 nph-regulr 70-30 mix 100 unit/ml subcutaneous susp  Insulin lispro (u-100) 100 unit/ml subcutaneous pen  Insulin lispro (u-100) 100 unit/ml subcutaneous solution  Insulin nph human recomb 100 unit/ml subcutaneous suspension  Insulin reg 1 unit/ml bolus from infusion (endotool)  Insulin regular 1 unit/ml 250 ml infusion  Insulin regular 1 unit/ml 250 ml infusion  Insulin regular 1 unit/ml infusion  Insulin regular human u-500concentrate" 500 unit/ml subcutaneous soln"  Isoproterenol infusion (rex)  Isoproterenol infusion (rex)  Ketamine (ketalar) 10 mg/ml injection  Ketamine (ketalar) 10 mg/ml injection  Ketamine 100 mg/100 ml ns infusion  Ketamine infn 250mg/250ml (1mg/ml) ns (50 mg/ml main ingredient)  Lactated ringers intravenous solution  Lactulose 10 gram/15 ml (15 ml) oral solution  Lactulose 10 gram/15 ml oral solution  Lactulose 20 g oral packet  Lactulose enema  Levofloxacin 250 mg tablet  Levofloxacin 500 mg tablet  Levofloxacin 750 mg tablet  Levofloxacin 750 mg/150 ml in 5 % dextrose intravenous piggyback  Lidocaine (cardiac) (xylocaine) 100 mg/5 ml (2 %) injection  Lidocaine (pf) 4 mg/ml (0.4 %) in 5 % dextrose intravenous solution  Linezolid 600 mg tablet  Linezolid 600 mg/300 ml intravenous solution  Lithium carbonate er 450 mg tablet,extended release  Lopinavir-ritonavir 200 mg-50 mg tablet  Lorazepam (ativan) 2 mg/ml injection  Lorazepam 0.5 mg tablet  Lorazepam 1 mg tablet  Lorazepam 2 mg tablet  Lorazepam 2 mg/ml injection solution  Lorazepam 2 mg/ml oral concentrate  Lovenox subq  Mannitol 25 % injection  Meperidine (demerol) 25 mg/ml injection  Meperidine (pf) 25 mg/ml injection wrapper  Meropenem 1000 mg ivpb mbp (extended infusion)  Meropenem 500mg ivpb mbp (extended infusion)  Meropenem ivpb 1000 mg connector bag  Methadone 10 mg tablet  Methadone 5 mg tablet | Metronidazole 500 mg tablet  Metronidazole 500 mg/100 ml-sodium chloride(iso) intravenous piggyback  Micafungin ivpb (50 mg vial) in 100 ml  Midazolam 1 mg/ml (combined) injection solution  Midazolam 1 mg/ml in 0.9 % sodium chloride intravenous  Midazolam 2 mg/ml syrup  Midazolam 5 mg/ml injection solution  Midodrine 2.5 mg tablet  Midodrine 2.5 mg tablet  Mineral oil (30 ml) oral oil  Minocycline 100 mg capsule  Mirtazapine 15 mg tablet  Morphine (pf) 1 mg/ml injection solution  Morphine (pf) 2 mg/ml 2 mg/ml injection  Morphine (pf) 4 mg/ml injection  Morphine 1 mg/ml in 0.9 % sodium chloride intravenous  Morphine 1 mg/ml in dextrose 5 % injection  Morphine 10 mg/ml inj combined superorderable  Morphine 2 mg/ml injection pf wrapper  Morphine 4 mg/ml injection  Morphine concentrate 100 mg/5 ml (20 mg/ml) oral solution  Morphine er 15 mg tablet,extended release  Morphine infusion 1 mg/ml (10 mg/ml vial)  Mycophenolate mofetil 200 mg/ml oral suspension  Mycophenolate mofetil 500 mg tablet  Naloxone infusion 16 mcg/ml in 250 ml  Nicardipine infusion 0.1 mg/ml in 250 ml  Nitroglycerin 100 mg/250 ml (400 mcg/ml) in 5 % dextrose intravenous  Nitroglycerin 50 mg/250 ml (200 mcg/ml) in 5 % dextrose intravenous  Nitroglycerin 50mg/d5w 250 ml (200 mcg/ml) premix 50 mg/250 ml (200 mcg/ml) infusion  Nitroprusside 50 mg/100 ml (500 mcg/ml) in d5w infusion  Nitroprusside infusion (adult)  Norepinephrine bitartrate 8 mg/250 ml (32 mcg/ml) in 0.9 % nacl iv  Norepinephrine bitartrate-ns 8 mg/250 ml (32 mcg/ml) infusion  Norepinephrine infusion in 250 ml  Norepinephrine infusion in 250 ml (unc)  Octreotide 500 mcg/100 ml (5 mcg/ml) in ns infusion  Octreotide acetate 100 mcg/ml injection solution  Octreotide iv infusion  Octreotide iv infusion  Omeprazole 2 mg/ml oral suspension  Omeprazole 20 mg capsule,delayed release  Omeprazole 40 mg capsule,delayed release  Oseltamivir 30 mg capsule  Oseltamivir 75 mg capsule  Osimertinib 80 mg tablet  Oxacillin 2 gram/50 ml in dextrose (iso-osmotic) intravenous piggyback  Oxcarbazepine 300 mg tablet  Oxycodone (roxicodone) 5 mg immediate release tablet  Oxycodone (roxicodone) 5 mg/5 ml solution  Oxycodone 15 mg tablet  Oxycodone 20 mg tablet |
| Oxycodone 5 mg tablet,oral only (not feeding tubes)  Oxycodone er 30 mg tablet,crush resistant,extended release 12 hr  Oxycodone-acetaminophen 5 mg-325 mg tablet  Pantoprazole 20 mg tablet,delayed release  Pantoprazole dr 40 mg granules delayed-release for susp in packet  Peg 3350-electrolytes 236 gram-22.74 gram-6.74 gram-5.86 gram solution  Pentamidine ivpb in 100 ml  Pentamidine ivpb in 100 ml  Pentobarbital 2500mg/50 ml adult infusion  Phenylephrine (pf) 20 mg/250 ml (80 mcg/ml) in 0.9% sodium chloride iv  Phenytoin maintenance ivpb  Phenytoin sodium extended 100 mg capsule  Piperacillin-tazobactam 2.25 gm / 50 ml (traditional)  Piperacillin-tazobactam 2.25 gram/50 ml in dextrose(iso) iv piggyback  Piperacillin-tazobactam 3.375 g 100ml mini-bag plus  Piperacillin-tazobactam 4.5 g mini-bag plus  Piperacillin-tazobactam 4.5 gram/100 ml dextrose(iso-osm) iv piggyback  Polyethylene glycol 3350 17 gram oral powder packet  Posaconazole ivpb in 100ml  Propofol (diprivan) 10 mg/ml injection  Propofol infusion 10 mg/ml  Prothrombin complex (kcentra) intermittent infusion  Psyllium husk (aspartame) 3.4 gram oral powder packet  Raltegravir 400 mg tablet  Remdesivir in ns 250 ml ivpb (pwdr)  Remdesivir in ns 250 ml ivpb (soln)  Remifentanil (ultiva) 1 mg injection  Rifampin 300 mg capsule  Rivaroxaban 15 mg tablet  Rivaroxaban 20 mg tablet  Rocuronium (zemuron) 10 mg/ml injection  Rocuronium 50 mg/5 ml (10 mg/ml) syringe  Sennosides 8.6 mg-docusate sodium 50 mg tablet  Sennosides 8.8 mg/5 ml oral syrup  Sodium bicarbonate continuous infusion  Sodium bicarbonate continuous infusion (cldh)  Sodium bicarbonate infusion in ns  Sodium chloride (ns) 0.9 % infusion  Sodium chloride 0.45 % intravenous solution  Sodium chloride 0.9 % intravenous solution  Sodium chloride 0.9 % iv bolus (cath lab)  Sodium chloride 1.5 % infusion  Sodium chloride 1.5 % infusion  Sodium chloride 3 % intravenous bolus solution  Sodium chloride 4 meq/ml 23.4% injection  Sodium nitroprusside (nipride) 25 mg/ml injection  Sodium phosphates 19 gram-7 gram/118 ml enema  Sotalol 160 mg tablet  Sotalol 80 mg tablet  Succinylcholine (anectine) 20 mg/ml injection  Succinylcholine chloride (anectine) 100 mg/5 ml (20 mg/ml) injection | Succinylcholine chloride 20mg/ml syringe/vial wrapper  Sugammadex (bridion) 100 mg/ml injection  Sulfamethoxazole 200 mg-trimethoprim 40 mg/5 ml oral suspension  Sulfamethoxazole 800 mg-trimethoprim 160 mg tablet  Tacrolimus 0.5 mg capsule  Tacrolimus 1 mg capsule  Theophylline 80 mg/15 ml oral elixir  Theophylline er 200 mg tablet,extended release,12 hr  Tobramycin ivpb in 100 ml  Tocilizumab (actemra) ivpb in 100 ml  Tramadol 50 mg tablet  Valacyclovir 500 mg tablet  Valganciclovir 450 mg tablet  Valproic acid ivpb in 100 ml  Vancomycin (vancocin) 1,000 mg injection  Vancomycin 1 gram/200 ml in dextrose 5 % intravenous piggyback  Vancomycin 1.25 gram/250 ml in 0.9 % sodium chloride intravenous  Vancomycin 1.75 gram/500 ml in 0.9 % sodium chloride intravenous  Vancomycin 125 mg capsule  Vancomycin 1500 mg in 500 ml ivpb  Vancomycin 1750 mg in 500 ml ivpb  Vancomycin 2 gram/500 ml in 0.9 % sodium chloride intravenous  Vancomycin 2000 mg in 500 ml ivpb  Vancomycin 25 mg/ml oral solution  Vancomycin 2500 mg in 500 ml ivpb  Vancomycin 50 mg/ml oral solution  Vancomycin 500 mg in 100 ml ivpb connector bag  Vancomycin 750 mg in 250 ml ivpb  Vancomycin fluid restricted (<1 g doses) iv infusion  Vancomycin in 1000 ml ivpb  Vancomycin ivpb in 250 ml  Vancomycin ivpb in 500 ml  Vasopressin (pitressin) 40 units/50 ml (0.08 unit/ml) infusion  Vasopressin (pitressin) infusion 40 units/100 ml  Vasopressin (pitressin) infusion 40 units/100 ml  Vasopressin 40 units/50 ml (0.8 unit/ml) in ns infusion  Vecuronium bromide 20 mg intravenous solution  Vecuronium in sterile water 10 mg/10 ml (1 mg/ml)  Vecuronium infusion 100 mg/100 ml (1 mg/ml)  Voriconazole 200 mg tablet  Voriconazole ivpb in 250 ml (loading)  Warfarin 1 mg tablet  Warfarin 2.5 mg tablet  Warfarin 3 mg tablet  Warfarin 4 mg tablet  Warfarin 5 mg tablet |  |
